# Copy Number Variation of the Beta-Defensin Genes in Europeans: No Supporting Evidence for Association with Lung Function, Chronic Obstructive Pulmonary Disease or Asthma

**DOI:** 10.1371/journal.pone.0084192

**Published:** 2014-01-03

**Authors:** Louise V. Wain, Linda Odenthal-Hesse, Razan Abujaber, Ian Sayers, Caroline Beardsmore, Erol A. Gaillard, Sally Chappell, Cristian M. Dogaru, Tricia McKeever, Tamar Guetta-Baranes, Noor Kalsheker, Claudia E. Kuehni, Ian P. Hall, Martin D. Tobin, Edward J. Hollox

**Affiliations:** 1 Department of Health Sciences, University of Leicester, Leicester, United Kingdom; 2 Department of Genetics, University of Leicester, Leicester, United Kingdom; 3 Division of Respiratory Medicine, School of Medicine, University of Nottingham, Nottingham, United Kingdom; 4 Department of Infection, Immunity and Inflammation, University of Leicester, Leicester, United Kingdom; 5 Institute for Lung Health, National Institute for Health Research (NIHR) Leicester Respiratory Biomedical Research Unit, Glenfield Hospital, Leicester, United Kingdom; 6 School of Life Sciences, University of Nottingham, Nottingham, United Kingdom; 7 Institute of Social and Preventive Medicine, University of Bern, Bern, Switzerland; 8 School of Community Health Sciences, University of Nottingham, Nottingham, United Kingdom; South Texas Veterans Health Care System and University Health Science Center San Antonio, United States of America

## Abstract

Lung function measures are heritable, predict mortality and are relevant in diagnosis of chronic obstructive pulmonary disease (COPD). COPD and asthma are diseases of the airways with major public health impacts and each have a heritable component. Genome-wide association studies of SNPs have revealed novel genetic associations with both diseases but only account for a small proportion of the heritability. Complex copy number variation may account for some of the missing heritability. A well-characterised genomic region of complex copy number variation contains beta-defensin genes (*DEFB103*, *DEFB104* and *DEFB4*), which have a role in the innate immune response. Previous studies have implicated these and related genes as being associated with asthma or COPD. We hypothesised that copy number variation of these genes may play a role in lung function in the general population and in COPD and asthma risk. We undertook copy number typing of this locus in 1149 adult and 689 children using a paralogue ratio test and investigated association with COPD, asthma and lung function. Replication of findings was assessed in a larger independent sample of COPD cases and smoking controls. We found evidence for an association of beta-defensin copy number with COPD in the adult cohort (OR = 1.4, 95%CI:1.02–1.92, P = 0.039) but this finding, and findings from a previous study, were not replicated in a larger follow-up sample(OR = 0.89, 95%CI:0.72–1.07, P = 0.217). No robust evidence of association with asthma in children was observed. We found no evidence for association between beta-defensin copy number and lung function in the general populations. Our findings suggest that previous reports of association of beta-defensin copy number with COPD should be viewed with caution. Suboptimal measurement of copy number can lead to spurious associations. Further beta-defensin copy number measurement in larger sample sizes of COPD cases and children with asthma are needed.

## Introduction

Lung function measures are heritable traits that predict morbidity and mortality in the general population [1]–[4]. The ratio of forced expired volume in 1 second (FEV_1_) to forced vital capacity (FVC) is used in diagnostic criteria for Chronic obstructive pulmonary disease (COPD), whilst the FEV_1_ (expressed as % predicted FEV_1_) contributes to measures of COPD severity. Genome-wide association studies (GWAS) of single nucleotide polymorphisms (SNPs) have identified 26 regions of the genome showing association with FEV_1_ and/or FEV_1_/FVC [5]–[7]. Collectively these 26 variants only account for approximately 3.2% of the additive polygenic variance in FEV_1_/FVC [7]. COPD, characterised by irreversible airway obstruction, is a leading cause of global mortality and morbidity predicted to be the third leading cause of death by 2020 [8]. Although the major risk factor for COPD is smoking, there is a heritable component [9]. We previously hypothesised that some the genetic determinants of lung function would be genetic causes of COPD. Of the 26 genomic regions associated with lung function, 8 have so far been reported to be associated with airway obstruction or COPD [10]–[14].

Asthma, a form of reversible airway obstruction, has increased in prevalence and more than 300 million people worldwide are affected [15]. Whilst GWAS for asthma have shown association with at least 10 genomic regions, including those encoding proteins involved in the immune response [16]–[19], these variants explain only around 4% of asthma heritability [20]. Thus, most of the genetic determinants of lung function, COPD and asthma are yet to be identified. Copy number variation (CNV) is a type of genetic variation where sections of the genome are duplicated or deleted. It is currently estimated that up to 4% of the genome may be copy number variable [21]. Copy number variation, particularly complex copy number variation which is poorly tagged by the variants commonly studied in GWAS, may explain some of the remaining unexplained heritability of these clinically important traits.

One of the best-characterised regions of complex copy number variation is the beta-defensin locus; the repeat unit (referred to hereafter as DEFB) contains several beta defensin genes; *DEFB107*, *DEFB106*, *DEFB105*, *DEFB104*, *DEFB103* and *DEFB4* and also *SPAG11*, which vary in copy number as a block. In addition to their direct antimicrobial function, these beta-defensins can act as chemokines having an immunomodulatory role (for review see [22]). Both *DEFB4* and *DEFB103* are expressed in the lung and trachea [23], [24], and the mature peptides hbd2 (encoded by the gene *DEFB4*) and hbd3 (encoded by the gene *DEFB103*) have antimicrobial activity against a range of pathogens that are known to infect the airway [25]. The mature peptide hbd4 (encoded by the gene *DEFB104*) is also present in appreciable quantities in the lung [26], and expression of these three beta-defensins are modulated by pro-inflammatory molecules or bacteria [27], [28]. In the UK population, DEFB copy numbers of between 2 and 10 are observed at an appreciable frequency [29], [30]. Thus there has been considerable interest in relating DEFB copy number to immune-related diseases including COPD and asthma, as well as psoriasis [31], [32]. However, the findings from association studies of other diseases have not always been consistent and replicable [33]–[36].

We determined DEFB diploid copy number using a Paralogue Ratio Test (PRT), a method shown to have higher accuracy than previously employed methods [29], in a total of 2343 individuals from two UK-based general population cohorts of adults and children (Table 1 and Table 2). We tested association firstly with quantitative measures of lung function in all individuals (FEV_1_, FVC and FEV_1_/FVC) and with risk of asthma and risk of COPD in subsets of each cohort.

**Table 1:** Characteristics of Gedling and LRC individuals included in the study.

	LRC (n = 689)	Gedling (n = 1149)
Age (mean[range])	12.8 [7.8–14.2]	56.1 [27.5–80.1]
Sex (males:females)	354∶335	569∶580
Height cm (mean[range])	156.4 [122.6–187.7]	168.0 [145.0–204.0]
FEV_1_ litres (mean[range])	2.70 [1.17–4.71]	2.86 [0.51–5.81]
FVC litres (mean[range])	3.08 [1.43–5.84]	3.68 [0.80–7.13]
FEV_1_/FVC (mean[range])	0.88 [0.64–0.997]	0.77 [0.35–0.96]
Doctor diagnosed asthma (yes:no)	255∶434	45∶629*
Asthma-ICS	82∶441	na
COPD GOLD Stage 2	na	42∶189^#^

Individuals with % predicted FEV_1_<80% and FEV_1_/FVC<0.7 (GOLD stage 2 cases) excluded from cases and controls. ^#^Characteristics of cases and controls are shown in Table 2.

**Table 2:** Characteristics of Gedling and replication COPD cases and controls.

	Gedling		Replication	
	Cases (n = 42)	Controls (n = 189)	Cases (n = 251)	Controls (n = 227)
Age (mean[range])	67.4[45–80]	59.4[41–80]	66.8[35–90]	57.0[38–88]
Sex (Males:Females)	31∶11	119∶70	142∶109	95∶132
Height (mean[range])	168.3[145–182]	168.8[146–190]	166.1[148–188]	167.1[146–190]
FEV_1_ (mean[range])	1.72[0.7–2.93]	2.97[1.65–5.14]	1.01[0.25–2.53]	2.80[1.38–4.83]]
% predicted FEV_1_ (mean[range])	60.9[24.0–79.9]	97.3[80.2–151.7]	37.8[11.2–77.2]	93.5[80.0–127.7]
FVC (mean[range])	2.79[1.36–4.32]	3.78[2.15–9.65]	2.20[0.82–4.17]	3.52[1.64–6.61]
FEV_1_/FVC (mean[range])	0.61[0.35–0.70]	0.79[0.70–0.92]	0.46[0.13–0.69]	0.79[0.71–0.97]
Smoking pack years (mean[range])	42.7[6–150]	22.5[5–90]	46.6[20–150]	35.6[20–145]

## Materials and Methods

### Ethics Statement

The Gedling study was approved by the Nottingham City Hospital and Nottingham University Ethics committees (MREC/99/4/01) and written informed consent for genetic study was obtained from participants. The Leicester Respiratory Cohort was approved by the Leicestershire Health Authority Research Ethics Committee, Leicester, UK (07/H0407/70) and written informed consent for genetic study was obtained from the subjects and their parents. For the replication COPD cases and controls, approval was obtained from Southmead Local Research Ethics Committee, Bristol (134/01), and Lothian Research Ethics Committee, Edinburgh (LREC/2001/4/62) and written informed consent for genetic study was obtained from participants.

### Sample selection

#### Gedling

**Figure 1:**
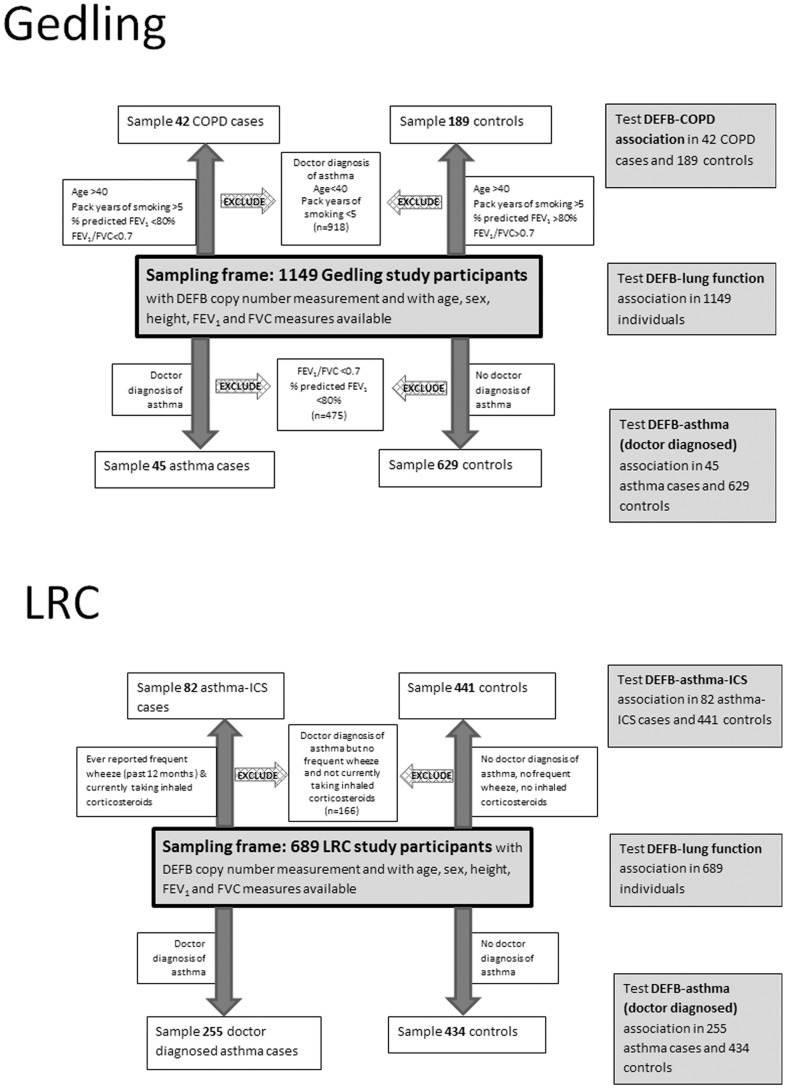
Sample selection for association analyses in Gedling and LRC. The sampling frame for each cohort is shown centrally with the COPD and asthma association analysis sample subsets indicated above and below for each cohort.

The Gedling cohort is a general population sample recruited in Nottingham in 1991 (18 to 70 years of age, n = 2,633) [37] and then followed-up in 2000 (n = 1346) when blood samples were taken for DNA extraction and FEV_1_ and FVC were measured using a calibrated dry bellows spirometer (Vitalograph, Buckingham, UK), recording the best of three satisfactory attempts [38]. DNA was extracted by Source Biosciences, UK. A total of 1176 European ancestry individuals for whom DNA and non-missing relevant phenotype data (age, sex, height, FEV_1_ (pre-bronchodilator), FVC (pre-bronchodilator), smoking status, asthma diagnosis) were available and were typed for DEFB copy number using PRT. Doctor diagnosed asthma cases in Gedling were defined as individuals for whom an asthma diagnosis had been confirmed by a doctor [37] (however, individuals with % predicted FEV_1_<80% and FEV_1_/FVC<0.7 (COPD GOLD stage 2 [39]) were excluded from both doctor diagnosed asthma case and control sets. Percent predicted FEV_1_ was calculated according to previously described equations [40], [41]). COPD cases were defined in Gedling as individuals age>40, smoking pack years>5, % predicted FEV_1_<80% and FEV_1_/FVC<0.7 (COPD GOLD stage 2 and above [39]). Controls for the COPD analysis were defined as age>40, pack years >5, %predicted FEV_1_>80% and FEV_1_/FVC>0.7. Individuals with a doctor diagnosis of asthma were excluded from the COPD case and control sets. Characteristics of these samples are given in Table 1 and Table 2. Figure 1 (top) illustrates how samples were selected for each analysis.

#### Leicester Respiratory Cohort (LRC)

The paediatric dataset comes from a population-based respiratory cohort in Leicestershire, UK, described in detail elsewhere [42]. In short, perinatal data were collected at birth, and data on growth and development were acquired prospectively during childhood. Respiratory morbidity and individual and family-related exposures were assessed by repeated questionnaires (1998, 2001, 2003, 2006, 2010). In 2006 to 2010, families who had returned two or more questionnaires (n = 4,125) were invited to the laboratory for assessment of lung function by spirometry [43], [44]. We recorded FVC and FEV_1_ before and 15 minutes after administration of salbutamol (400 µg) by spacer. Pre-bronchodilator measurements were used in this study. Of 4,177 children who attended the lab, 903 individuals had saliva samples available for DNA extraction. DNA was extracted by LGC Genomics Ltd (previously KBiosciences Ltd). Of these, 689 were of European ancestry (self-reported), aged under 16 years and had non-missing relevant phenotype data (age, sex, height, FEV_1_, FVC, and asthma variables). The LRC is an unselected population-based cohort and most children reporting a doctor diagnosis of asthma had mild disease. Therefore, a potentially more clinically relevant subpopulation with moderately severe asthma was also defined as individuals who had ever reported frequent wheeze during the past 12 months (over four attacks or always accompanied by shortness of breath) and were taking inhaled corticosteroids at the time of questionnaire (controls for this analysis were all individuals who had never reported frequent wheeze during the past 12 months, were not taking inhaled corticosteroids at the time of questionnaire and did not report a doctor diagnosis of asthma). This phenotype is referred to hereafter as “asthma-ICS”. Characteristics of LRC subjects are given in Table 1. Figure 1 (bottom) illustrates how samples were selected for each analysis.

#### COPD replication collection

The criteria for recruitment of cases and controls in the replication collection are described elsewhere [45]. In brief, the criteria for cases was % predicted FEV_1_< = 70%, FEV_1_/FVC<70%, no significant reversibility on bronchodilation, a smoking history of > = 20 pack years, and no diagnosis of asthma or other alternative diagnosis for airway obstruction (including severe SERPINA1 deficiency). Controls were selected who had no history of airway obstruction or recent pulmonary infection, and had % predicted FEV_1_> = 80% and FEV_1_/FVC>70% and were selected to match the cases for age and sex. Controls were current or ex-smokers with smoking history similar to that of the cases. A total of 251 UK cases (112 from Bristol and 139 from Edinburgh) and 227 UK controls (136 from Bristol and 91 from Edinburgh) were copy number genotyped for DEFB copy number using PRT in the same laboratory as the Gedling and LRC samples. Cases and controls were randomised and the individual undertaking the assays was blind to COPD status. All individuals were of European ancestry. Characteristics of these samples are given in Table 2.

### DEFB copy number assay

The DEFB copy number was determined, blind to clinical information, using a triplex PRT described previously [29], [46]. Every experiment included the same six positive controls of known copy number, the same samples as used in previous studies [29], [30], which were used to normalize the experimental results and minimize interexperimental technical variation in typing copy number. Maximum likelihood integer copy number was called by combining information from all three assays and estimating the most likely copy number given the data. In addition, non-integer copy number values were calculated by averaging the estimates from the three assays.

### Statistical analysis

Association of the average raw PRT DEFB copy numbers with lung function in all individuals (FEV_1_, FVC and FEV_1_/FVC), COPD status (Gedling only) and asthma status (doctor diagnosed asthma in both cohorts and asthma-ICS in LRC) was tested using linear and logistic regression. Association of the maximum likelihood integer copy numbers with lung function, COPD status and asthma status was also undertaken. Age, age^2^, sex and height were included as covariates. Inverse normal transformation was applied to FEV_1_ and FEV_1_/FVC. R v2.15.2 was used to run all association analyses. Power was estimated by a simulated study of equivalent size to the Gedling study (n = 1149) with a similar distribution of DEFB copy number to that observed in our data. At a significance level of 0.05, power was 95% to detect an effect size of 0.1 SD change in lung function per one unit change in DEFB copy number. In 42 COPD cases and 189 controls, simulations showed, for a significance level of 0.05, power of 88% to detect an OR of 1.7 per unit change in DEFB copy number. Power simulations were run using Stata version 13.

### Replication analysis

A subset of a larger European ancestry COPD case-control study was used to test for replication of an association with COPD observed in the Gedling cohort. Logistic regression was used to test for association with COPD using age, age^2^, sex and height as covariates in the model. Comparison of distributions of copy numbers was undertaken using a Mann-Whitney-Wilcoxon test and comparison of frequencies of samples with copy number > = 5 in cases and controls was undertaken using a Pearson's chi-squared test (undertaken using R v2.15.2).

## Results

**Figure 2:**
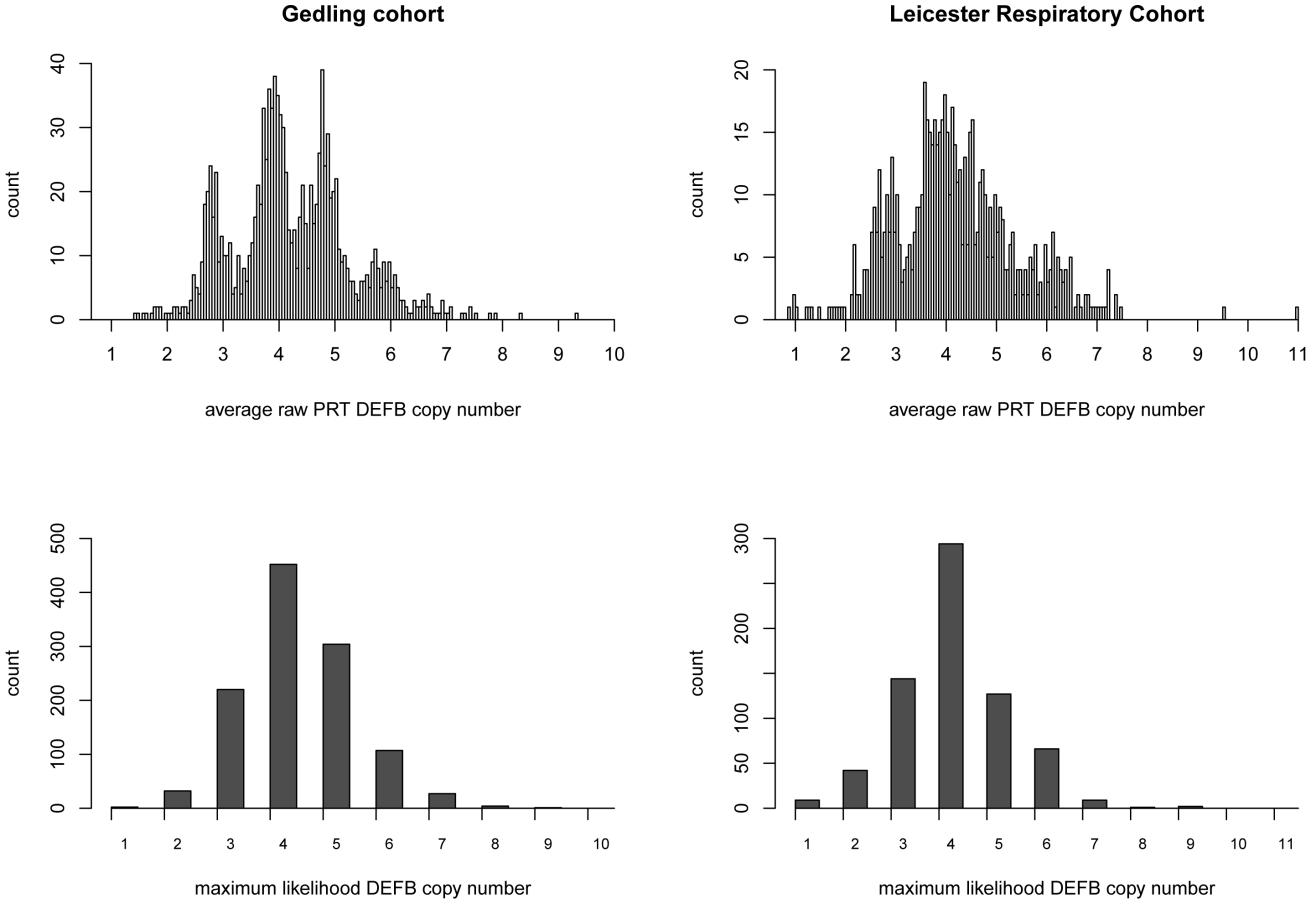
Distributions of raw PRT copy number (top) and maximum likelihod integer copy number (bottom) for Gedling (right) and LRC (left) cohorts.

DEFB copy number was successfully measured using PRT in 689 LRC children and 1149 Gedling adults (Table 1). The distributions of raw PRT copy numbers and maximum likelihood integer copy numbers for each cohort are shown in Figure 2. In LRC, maximum likelihood integer copy numbers ranged from 1 to 9 with a mean copy number of 4.06. In the Gedling population, copy numbers ranged from 1 to 9 with a mean of 4.3. Association of DEFB raw PRT copy number and lung function (FEV_1_, FVC and FEV_1_/FVC), COPD (Gedling only) and asthma was tested separately in each cohort and the results are presented in Table 3. Association of maximum likelihood integer copy numbers of DEFB with all traits were also undertaken and the results were consistent with those obtained using the raw PRT copy numbers (results not shown).

**Table 3:** Association of DEFB with lung function, asthma and COPD in LRC and Gedling.

	LRC				Gedling			
Lung function (inverse normal transformed)	**N**	**Beta**	**SE**	**P**	**N**	**Beta**	**SE**	**P**
FEV_1_	689	0.019	0.020	0.333	1149	−0.026	0.016	0.114
FVC	689	0.014	0.020	0.490	1149	−0.008	0.015	0.581
FEV_1_/FVC	689	0.019	0.030	0.525	1149	−0.043	0.025	0.090
FEV_1_/FVC - never smokers only					647	−0.036	0.031	0.252
FEV_1_/FVC - ever smokers only					502	−0.067	0.0417	0.106
**Asthma**	**N (case:control)^#^**	**OR**	**CI**	**P**	**N (case:control)^#^**	**OR**	**CI**	**P**
Doctor diagnosed asthma	255∶434	1.08	0.95–1.23	0.244	45∶629	1.03	0.79–1.37	0.784
Asthma-ICS	82∶441	1.26	1.07–1.54	0.016*				
**COPD**					**N (case:control)^#^**	**OR**	**CI**	**P**
COPD GOLD Stage 2					42∶189	1.40	1.02–1.92	0.039

P = 0.043 if maximum likelihood DEFB integer copy numbers used ^#^controls for each analysis as defined in the method.

**Figure 3:**
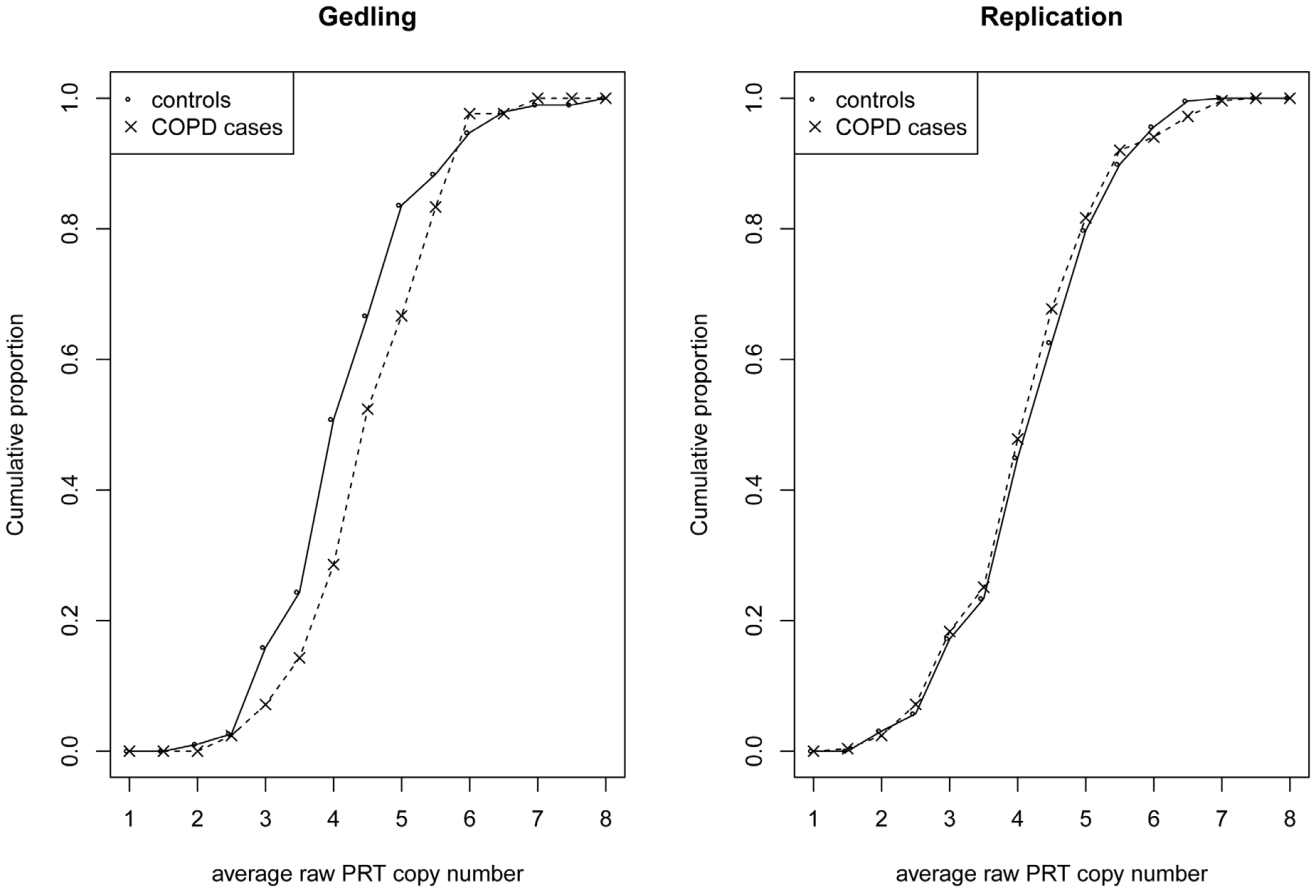
Cumulative frequency distribution of average raw PRT DEFB copy number in Gedling COPD cases and controls (left) and replication COPD cases and controls (right).

Although there was no evidence for association of DEFB copy number with lung function in all individuals in either cohort (even when the data for adults were stratified by smoking status), a significant (P<0.05) signal of association of DEFB copy number with COPD was observed within the adult Gedling population (OR = 1.34, 95% CI:1.02–1.77, P = 0.039) (Figure 3). When we sought replication in a sample of 251 UK COPD cases and 227 UK controls [45], there was no evidence for association of DEFB copy number and COPD (OR = 0.89, 95% CI:0.72–1.07, P = 0.217) (Figure 3). In fact, we found a trend for an association in the opposite direction (increased copy number associated with decreased risk of COPD) to that observed in the Gedling population.

**Figure 4:**
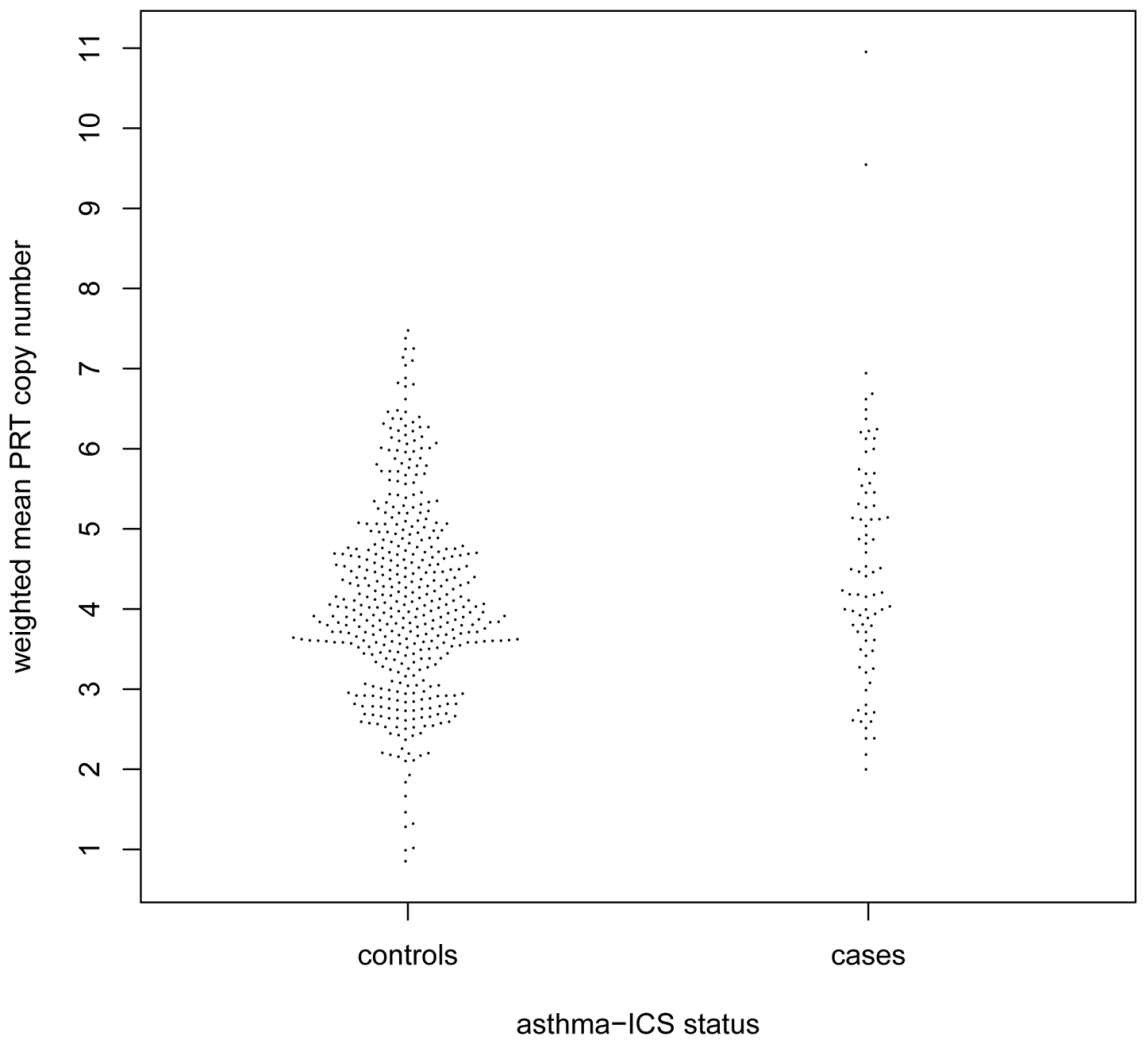
DEFB copy number in LRC asthma-ICS cases and controls.

We did not observe evidence for association of DEFB copy number with doctor-diagnosed asthma for either LRC or Gedling. Because the majority of the children with doctor-diagnosed asthma in LRC had very mild disease, we also used a stricter definition of clinically relevant asthma (asthma-ICS) in LRC. We found a significant association of DEFB copy number with asthma-ICS for LRC (OR = 1.26, 95% CI:1.07–1.54, P = 0.016). However, a comparison of the distributions of DEFB copy number in the asthma-ICS cases and controls showed that the signal was driven by 2 cases with high copy numbers (Figure 4). After exclusion of these two individuals the strength of the association decreased considerably (OR = 1.18, 95% CI: 0.96–1.44, P = 0.12).

## Discussion

This study determined DEFB diploid copy number in a cohort of 1149 adults and in a separate cohort of 689 children using PRT, which is currently the most accurate method for measuring copy number variation at this locus. The distributions of copy number observed in each cohort were in good agreement with previously published distributions from the UK population [29], [47], although in the LRC cohort we observed nine children (>1%) with a copy number of 1, which in other cohorts is rare. We tested for association with lung function as a quantitative measurement (FEV_1_ and FEV_1_/FVC) in all individuals in each cohort as well as for association with COPD and asthma using appropriate case and control subsets of individuals within each cohort. Previous studies have reported some evidence for association of other beta-defensins with COPD [48], [49] and asthma [50]. However, our study, using a reliable measure of DEFB (comprising *DEFB4*, *DEFB103* and *DEFB104* genes) copy number, does not show a strong effect of DEFB copy number on these traits. In addition, we found no evidence for association with lung function in the general population.

A previous study presented evidence of an association of higher DEFB copy number with increased expression levels in nasal epithelial cells and with a higher risk of COPD [51]. Janssens et al [51] used real time quantitative PCR to measure diploid DEFB copy number and test for association with COPD in 204 COPD cases and 154 healthy smoker controls. A significant association was observed with the cases having a significantly higher median copy number than the controls (median of 5 in cases, 4 in controls, P<0.0001). In our replication collection of 251 cases and 227 controls, the median copy number was not significantly different in cases and controls (median of 4.05 in cases, 4.12 in controls, P = 0.316). Janssens *et al* showed a significant difference in the proportion of individuals with a copy number > = 5 in the COPD cases compared to the control group (52% and 38%, respectively, P = 0.008). However, in our replication collection, an opposite effect was seen with 18% of cases having copy number > = 5 and 20% of controls having copy number > = 5 (P = 0.67). We found no significant association between high copy number (> = 5 copies) and COPD risk (OR = 0.88, 95% CI: 0.6–1.4).

We recognise that the small case and control sample subsets within the Gedling cohort would limit statistical power to detect association with COPD risk and that diagnosis of COPD in this cohort was not based on reversibility. Previous genome-wide significant SNP associations with COPD have given ORs in the order of 1.1 to 1.3 per copy of the risk allele [10], [13]. In our discovery sample set (42 cases and 189 controls) we would have had 88% power to detect an OR of 1.7 per unit change in DEFB4 copy number. Power would have been higher in the larger replication set. However, we were not able to replicate the findings of Janssens *et al* [51]. Non-replication of the COPD association identified in the Gedling cohort by our replication collection may have been due to differences in the distributions of lung function measures between the discovery and replication case collections. This may be explained by differences in the ascertainment strategy of cases in each collection (diagnosis was based on reversibility in the replication collection). Given that SNPs reported in genome-wide association studies as associated with COPD also show association with lung function in general population samples [10]–[14] one might have expected any CNV showing association with COPD to be associated with lung function. Genome-wide significant associations with effect sizes of 0.03 to 0.09 standard deviations from the mean per allele have been observed for GWAS of SNPs including tens of thousands of samples [6], [7]. We had 75% power to detect an effect size of 0.075 per one unit change in DEFB4 copy number in the Gedling cohort (n = 1149).

A significant signal of association of DEFB copy number with a clinically relevant definition of asthma (asthma-ICS) in LRC was found to be driven by two cases with high copy number. The raw results from each independent component of the triplex PRT assay (data not shown) were consistent suggesting that the high copy numbers were real for these two cases. Although exclusion of these two individuals weakened the signal, we cannot rule out that rare instances of very high copy number could be causative for asthma.

LRC recruitment included children of European and South Asian ancestry; we limited this study to individuals of European ancestry based on questionnaire data (for ease of comparison with Gedling). Although there are no large differences in DEFB copy number variation across the world, some subpopulations, including two from South Asia, have been found to have outlying distributions of DEFB copy number [30].

The high mutation rate and multiallelic nature of the DEFB copy number variation means it is poorly tagged by nearby SNP alleles and measurement based on SNP probe intensity data is inadequate [30], [52], [53]. In this study, we measured DEFB copy number using PRT which has been shown to have higher accuracy for calling copy number than real time qPCR [46]. PRT is a form of qPCR which can more reliably measure copy number, particularly higher diploid copy numbers, through the use of identical primers to target the copy number variable region and a two-copy reference region. This avoids the issue of differential primer binding which can be a feature of real-time qPCR copy number measurement. Inaccurate copy number measurement has resulted in erroneous association of copy number with disease from this and other complex copy number variable regions. The previously reported association of DEFB copy number with COPD, described in detail above and not replicated in this study, was based on real-time qPCR measurements [51].

In conclusion, this study does not support previous findings of an association of DEFB copy number variation with COPD. We suggest that previous associations may have been due to inaccurate copy number measurement. A strength of our study was the accurate method of measurement of copy number. Prior to 2008, candidate SNP-based association studies often reported inconsistent findings, however these problems have diminished with the very large sample sizes now commonly employed in genome-wide association studies. Accurate copy number typing of very large studies will be needed to confidently rule out any modest effect of DEFB copy number variation on lung function, COPD or asthma.
